# Porous polyethylene implant for skull base reconstruction in transsphenoidal surgery: a systematic literature review and institutional case series

**DOI:** 10.1007/s10143-025-04069-w

**Published:** 2026-01-19

**Authors:** Marco Galeazzi, Simona Serioli, Giorgia De Rosa, Ludovico Agostini, Federico Valeri, Alberto Benato, Sage Rahm, Murad Sultanov, Rosalinda Calandrelli, Sabrina Chiloiro, Giuseppe Maria Della Pepa, Mario Rigante, Liverana Lauretti, Alessandro Olivi, Francesco Doglietto, Pier Paolo Mattogno

**Affiliations:** 1 Department of Neurosurgery, Fondazione Policlinico Universitario Agostino Gemelli IRCCS, Largo Agostino Gemelli 8, Rome, 00168 Italy; 2 Department of Neurosurgery, Papa Giovanni XXIII Hospital, Bergamo, Italy; 3 Department of Neurosurgery, University of Alabama at Birmingham, Birmingham, AL USA; 4 Medicine and Surgery, Università Cattolica del Sacro Cuore, Rome, 00168 Italy; 5 Radiology and Neuroradiology Unit, Department of Imaging, Radiation Therapy and Hematology, Università Cattolica del Sacro Cuore, Fondazione Policlinico Universitario Agostino Gemelli IRCCS, Rome, 00168 Italy; 6 Pituitary Unit, Department of Endocrinology, Fondazione Policlinico Universitario Agostino Gemelli IRCCS, Rome, 00168 Italy; 7 Department of Aging, Neurological, Orthopedic and Head-Neck Sciences, Fondazione Policlinico Universitario Agostino Gemelli, Rome, 00168 Italy; 8 Department of Neurosurgery, Mater Olbia Hospital, Olbia, 07026 Italy

**Keywords:** Endoscopic endonasal surgery, Osteointegration, Porous polyethylene implant, Skull base reconstruction

## Abstract

Reconstruction of skull base (SB) defects following endoscopic endonasal approaches (EEA) is critical to minimize postoperative complications. Porous high-density polyethylene (Medpor^®^) implants have been employed for this purpose; however, their long-term complication rates and osteointegration outcomes remain unclear. A systematic review was conducted according to PRISMA guidelines across three databases, including studies involving SB reconstruction with porous polyethylene implants that reported complications and osteointegration outcomes. Additionally, a retrospective analysis was performed on 94 patients undergoing SB reconstruction in transsphenoidal surgery with Medpor (2008–2024). Osteointegration was assessed via imaging and clinical outcomes with a minimum follow-up of 1 year, as well as intraoperative findings in reoperated cases. Eleven studies (691 patients) were included, with reported postoperative complications including CSF leaks (2.9%), infections (2.3%), and rare implant extrusion or osteointegration failures (0.4%). In the institutional cohort, 25 of 94 patients (26.6%) required reoperation, with 17 undergoing reoperation at least 6 months post-implantation. All these patients were reoperated for pathology recurrence, except for one who was reoperated for spontaneous Medpor extrusion. Among these, 94% (16/17) had no evidence of osteointegration. Chronic nasal symptoms were reported in 13.4% of non-reoperated patients, with no cases requiring implant removal. One of the 17 reoperated patient had an additional surgery due to spontaneous Medpor Extrusion. The placement of the nasoseptal flap to cover Medpor did not appear to have a facilitating effect on its osseointegration; however, its role will need to be further studied. Medpor implants demonstrate acceptable long-term safety for EEA skull base reconstruction, with low rates of major complications. However, osteointegration appears infrequent (< 8%) even over extended follow-up.

## Introduction

Endoscopic skull base techniques have undergone significant advancements over recent decades, driven by innovations in high-definition imaging, neuronavigation, and customized microinstruments, thereby expanding surgical options for complex skull base pathologies [1]. Despite these advancements, the reconstruction of skull base (SB) defects following tumor resection remains a critical challenge in endoscopic skull base surgery [24].

Effective SB reconstruction should ensure durability, biocompatibility, low infection rates, and minimal inflammatory response while preventing complications such as cerebrospinal fluid (CSF) leaks, which remain the most common postoperative issue [24, 27]. Current reconstruction techniques utilize autologous materials (e.g., cartilage, bone grafts, and vascularized flaps) and synthetic substitutes, including bioabsorbable implants, titanium mesh, and dural sealants [2, 3, 14, 15, 17, 26, 27].

Porous high-density polyethylene (Medpor^®^) implants have been widely adopted in craniofacial and reconstructive surgeries to substitute the bony surface due to their ease of handling, biocompatibility, and structural support, and have seen increasing use in sellar floor repair during endoscopic endonasal approaches (EEA) [4, 8, 13, 19, 22, 23]. However, the long-term integration of Medpor with the surrounding bone, and particularly within the dynamic sinonasal environment, remains poorly characterized. Previous retrospective reports have described cases of chronic sphenoid sinusitis and implant extrusion requiring surgical removal, raising concerns about its long-term outcomes [12, 13].

A key unanswered question in the field is whether Medpor implants achieve osteointegration over time or merely function as inert structural supports within the skull base reconstruction. The lack of standardized methods to assess osteointegration, e.g., via imaging or intraoperative evaluation, has further limited clarity on this issue.

We hypothesized that Medpor implants, while providing acceptable clinical safety for EEA skull base reconstruction, would demonstrate low rates of true osteointegration even over long-term follow-up.

The primary aim of this study is to provide an updated evaluation of Medpor^®^-related complications and osteointegration in skull base reconstruction, integrating a systematic literature review with an institutional case series that evaluates radiological and intraoperative findings in patients undergoing reoperation after Medpor implantation. Understanding the behavior of Medpor in SB reconstruction is crucial for optimizing reconstructive strategies in EEA and guiding material selection in patients requiring long-term implant durability.

## Materials and methods

### Study design

This study integrates a systematic literature review and a retrospective institutional case series to evaluate porous high-density polyethylene (Medpor^®^) implants in endoscopic skull base (SB) reconstruction, focusing on long-term complication rates and osteointegration outcomes. The institutional case series is reported in accordance with the PROCESS guidelines [1].

### Systematic review

A comprehensive literature search was conducted in accordance with the PRISMA 2020 guidelines [21]; however, the review protocol was not registered. PubMed, Scopus, and Web of Science were searched on September 18, 2024, using combinations of the keywords: “Medpor”, “Complication*”, “Endoscopic Endonasal Approach”, “Transnasal Transsphenoidal Surgery”, “Skull base reconstruction”, “Extrusion”, and “Porous Polyethylene Implant”. No time frame restrictions were applied, and only English-language articles were considered.

Two independent reviewers (G.d.R., S.S.) screened titles and abstracts, followed by full-text assessments for eligibility. Disagreements were resolved through discussion with a third reviewer (M.G.).

Inclusion criteria were: (1) Studies reporting patients undergoing endoscopic SB reconstruction with porous polyethylene implants; (2) Reporting of postoperative complications (including CSF leak, infection, extrusion) related to Medpor^®^; (3) Availability of data on osteointegration during clinical or radiological follow-up. Exclusion criteria included reviews without new patient data, editorials, commentaries, and articles with incomplete data.

For each included study, the following data were extracted: first author, publication year, number of patients, gender distribution, underlying pathology, surgical approach, number of procedures, reconstruction method, use of Medpor^®^, postoperative complications, implant extrusion, osteointegration assessment, and follow-up duration.

### Institutional case series

Between February 2008 and November 2024, 1,874 patients underwent transsphenoidal surgery at the Fondazione Policlinico Universitario Agostino Gemelli IRCCS in Rome, Italy. Reconstruction of the sellar floor was a standard procedure in all cases and usually performed with bone or cartilage harvested from the nasal septum.

94 patients underwent SB reconstruction using Medpor implants (Medpor^®^ TSI 20 × 20 mm; Stryker Corporation, Kalamazoo, MI, USA).

Institutional practice at the time favored Medpor in: complex reconstructions, especially those involving extended skull-base approaches with significant bony defects; cases in which autologous bone or cartilage was unavailable or insufficient; selected revision cases or persistent CSF leaks in which previous autologous reinforcement of the sellar floor had failed.

In our institution, intraoperative CSF-leak management adheres to the Kelly et al. grading system. For grade 2 leaks and above, when autologous material was not available because of prior surgery, Medpor was employed as the rigid buttress, together with the application of sealant [7].

Patient Data Collection: Demographics, clinical history, histopathological diagnosis, imaging findings, surgical details, postoperative complications, and follow-up data (minimum 12 months) were collected retrospectively. For patients who underwent reoperation, operative reports and preoperative imaging were reviewed to assess implant osteointegration, defined as radiological evidence of bone incorporation or intraoperative findings of bony adherence or implant incorporation.

Inclusion for Osteointegration Analysis: Patients reoperated at least 6 months after Medpor placement were analyzed for osteointegration to allow sufficient time for potential integration.

Complication Assessment: Postoperative complications were categorized as early (< 30 days) and late (> 30 days), including CSF leaks, infections, chronic nasal symptoms, and implant-related issues (extrusion or removal).

For non-reoperated patients, structured follow-up was conducted using outpatient clinic visits and structured telephone interviews to assess nasal symptoms, potential reoperations at other centers, and long-term implant tolerance.

### Statistical analysis

Descriptive statistics were used to summarize patient demographics, complications, and outcomes. Categorical variables were presented as frequencies and percentages, while continuous variables were expressed as mean ± standard deviation (SD) or median with ranges, as appropriate. No inferential statistical analyses were planned due to the descriptive nature of the study.

### Ethics

All procedures were performed in accordance with the principles outlined in the Declaration of Helsinki. Institutional Review Board approval was obtained (IRB TYEPDD-6546), and informed consent was acquired from all patients for data collection and use.

## Results

### Systematic literature review

**Fig. 1:**
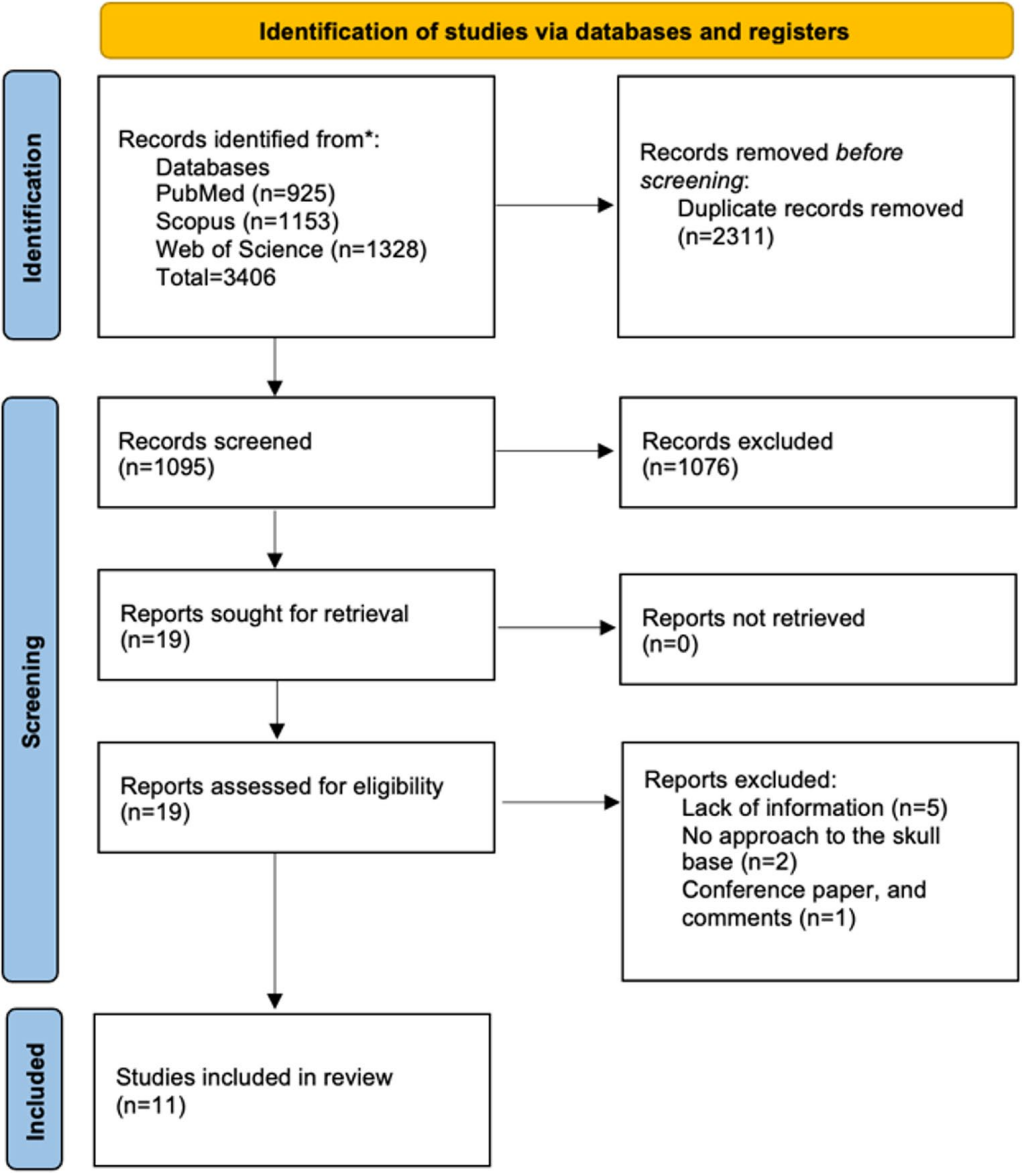
PRISMA flow diagram of the selection process

A total of 1095 articles were screened after duplicate removal, with 11 studies [9–13, 16, 18, 22, 25, 28, 30] meeting inclusion criteria and describing 691 patients who underwent transnasal skull base reconstruction with porous polyethylene implants (Figure 1).

The primary indications for surgery included pituitary neuroendocrine tumors (*n* = 415), CSF leaks (*n* = 77), craniopharyngiomas (*n* = 48), meningiomas (*n* = 45), and Rathke’s cleft cysts (*n* = 40). Reconstruction techniques varied across studies, with Medpor used alone or combined with adjuncts such as dural sealants, fat grafts, mucosal flaps, and injectable calcium phosphate cement (Table 1).

Postoperative complications potentially related to Medpor were reported in 5.7% (27/471) of patients for whom detailed data were available, and included CSF leaks (2.9%), infections (2.3%) - primarily sinusitis and meningitis – and rare cases of implant extrusion or osteointegration failure (0.4%) [9, 10, 12, 13, 18, 22, 25, 28]. In three patients, osteointegration failure was documented, with two identified radiologically on follow-up MRI [13] and one requiring implant removal due to chronic sphenoid sinusitis [12] (Table 1).

**Table 1:** A summary of the data extracted from the eleven studies included into the systematic literature review

First Author, Year of publication	*N*. of patients	Gender (M/F)	Pathology (*N*)	Surgical Approach	% of patients with Medpor	Type of reconstruction (*N*)	Post operative potentially Medpor related complications (*N*)	Failure of osteointegration (*N*)	Mean Follow-up (months Range)	Delayed Complications
Park, 2004 [22]	10	NA	NA	EEA/EEEA	100% (10)	Abdominal Fat + Medpor	No	No^ç^	16 months (9–23)	No
Wessell, 2013 [28]	5	3/2	CPh (2) TM (2) PM (1)	EEEA	100% (5)	Medpor + rectus sheath fascia	Aseptic meningitis (2) Sinusitis (2)	NA	10 months	
										NA
Hsu, 2015 [16]	9	5/4	I CSF leak (5) Tr. CSF leak (1) spontaneous (1) P-res. (2)	EEA/EEEA	33.3% (3/9)	Fat graft, bovine pericardium, Medpor, middle turbinate, and dural sealant (1) Fat graft, fascia lata, Medpor, and NS flap (1) Fat graft, fascia lata, Medpor, and dural sealant (1)	No	NA	8.7 months (2.9–19.3.9.3)	No
Liebelt, 2015 [18]	200	NA	PitNET (157) Chord. (1) Chondros. (1) RCC (29) Hyp. (4) AC (3) Ch. Hern (2) Germ. (1) Pneumoc. (1) PitC (1)	EEA combined with microscopic TSA	68% (136/200)	Fat graft + Medpor + dural sealant	Sinus irritation or drainage (6) CSF leak required operative re-exploration (1)	NA§	(12–60 months)	NA
Farhood, 2019 [12]	1	0/1	PitNET	EEA	100% (1)	Medpor	chronic sphenoid sinusitis (2 years after surgery)	Yes*	48 months	Reoperated to remove the Medpor implant
Farrell, 2019 [13]	139	70/69	PitNET (115) RCC (4) Other (20)	microscopic TSA: 135 EEA: 4	100% (139)	Medpor (73) Medpor + dural sealant: (52) Medpor + fat, fascia, duragen, dural sealant: (14)	NA	Yes ^ç^ (2)	26 months (0–105 months)	Chronic sinusitis (6) Medpor extrusion (2) Postnasal drip (1)
										Sphenoidotomy for Chronic Sphenoid Sinusitis: 5
Elarjani, 2020 [11]	1	1/0	Pm	EEEA	100% (1)	Fat graft + fascia lata + Medpor + NS flap	No	NA	2 months	No
Zhang, 2021 [30]	1	1/0	XGA	EEEA	100% (1)	Fat graft + fascia lata + Medpor + NS flap	No	NA	3 months	No
Cranial consortium, 2021 [9]	187	95/92	PitNET (142) CPh (13) RCC (7) M (4) Apoplexy (3) Chord (3) Other (15)	EEA: 134 Microscopic TSA: 25 EEEA: 28	1.60% (3/187)	NA	NA	NA	NA	NA
Smedley, 2024 [25]	68	NA	CSF leak	EEEA	100%	NA	CSF leak (7) Meningitis (3)	NA§	NA	NA
Desai, 2024 [10]	70	32/38	M (37) CPh (33)	EEEA	71.4% (50/70)	gasket seal with Medpor + NS flap (50)	CSF leak (6, 12%)	NA	NA	NA
Our Study	94	47/47	PitNETs (84) RCC (3) Chord. (3) M (2) CPh (1) SCO (1)	EEA/EEEA (87) Microscopic SLA (7)	100% (94)	Medpor, fat, duragen, and dural sealant: 16% Medpor and dural sealant: 23.5% Medpor and nasoseptal flap: 49.5% Flap, cartilage, fat, and medpor: 11%	No	Yes*^£^ (16/17) NA§	62.5 months (24–144 months)	Abscess (1) Extrusion (1)

AC, Arachnoid Cyst; Ch. Hern, Chiasmal Herniation; Chord., Chordoma; Chondros., Chondrosarcoma; CPh, Craniopharyngiomas; CSF, Cerebrospinal Fluid; EEA: Endoscopic Endonasal Approach; EEEA: Extended Endoscopic Endonasal Approach; FESS, Functional Endoscopic Sinus Surgery; Germ. Germinoma; Hyp., Hyperplasia; I, Iatrogenic; M, meningioma; NS, Nasoseptal, PitC, Pituitary Carcinoma; PitNETs, Pituitary Neuroendocrine Tumors; PM, Planum sphenoidale meningioma; Pneumoc., Pneumocephalus; P-res., Post-resection; RCC, Rathke cleft cysts; SCO, Spindle cell oncocytoma; SLA, Sublabial Approach; TM, Tuberculum meningioma; Tr. Traumatic; TSA, Transnasal Transphenoidal Surgery; XGA, Sellar xanhogranulomas, ^*^, Surgical evaluation of osteointegration; ^ç^, Radiological (MRI) evaluation of osteointegration; §, no extrusion; £, in 5 patients a sublabial microsurgical approach was performed at the first operation

### Institutional case series

Among the 94 patients (47 males, 47 females; mean age, 55.7 years; range, 17–86) who underwent sellar and parasellar reconstruction with Medpor during the study period, indications included pituitary neuroendocrine tumors (*n* = 84), Rathke’s cleft cysts (*n* = 3), chordomas (*n* = 3), meningiomas (*n* = 2), craniopharyngioma (*n* = 1), and spindle cell oncocytoma (*n* = 1).

Among the 77 patients who underwent a single operation, 7 (9.1%) were treated using a sublabial microsurgical approach, which did not involve the placement of a nasoseptal flap. Of the 17 patients who underwent reoperation at least 6 months later, 5 (29.4%) initially had surgery via the sublabial microsurgical route, followed by a second procedure using an EEA (Table 2).

**Table 2:** The table shows the clinical features of the 17 patients included in the study and reoperated at least after 6 months. All patients were reoperated for pathology recurrence, except patient n. 17 who was reoperated exclusively for Medpor extrusion. Although patients May have undergone multiple procedures, this table considers as the first intervention the one in which Medpor was placed, and as the last the one in which its state of osteointegration was assessed. The column “Previous surgery before Medpor placement” indicates whether the patient, prior to having Medpor positioned, underwent a previous surgery without Medpor placement. In the “Surgical Approach” column, (1) stands for microsurgical approach and (2) stands for EEA. Patients number 3, 5, 6, 10 and 13 underwent a first surgery through a microsurgical approach and a second surgery trough EEA. The column “evidence of osteointegration” refers to both intraoperative and radiological findings. Sealant was used in all cases for final reconstruction

*N*° Pts	Gender	Age	Pathology	Previous surgery before Medpor placement	Surgical Approach	Months between first and last surgery	Flap at previous surgery	Evidence of osteointegration	Medpor removed for final reconstruction	Flap in final reconstruction
1	M	47	NF- PitNET	No	2	81	No	No	Yes	No
2	F	46	NF-PitNET	Yes	2	75	No	No	No	No
3	F	57	Cordoma	Yes	First: 1 Second: 2	102	No	No	No	No
4	M	56	NF-PitNET	No	2	82	Yes	Yes	Yes	Yes
5	F	54	ACTH secreting-PitNET	Yes	First: 1 Second: 2	20	No	No	No	Yes
6	M	44	NF-PitNET	Yes	First: 1 Second: 2	23	No	No	Yes	Yes
7	F	61	NF-PitNET	Yes	2	65	No	No	Yes	Yes
8	M	60	NF-PitNET	No	2	85	No	No	Yes	No
9	F	60	NF-PitNET	Yes	2	64	Yes	No	Yes	Yes
10	F	61	NF-PitNET	Yes	First: 1 Second: 2	30	No	No	No	No
11	F	61	NF-PitNET	Yes	2	117	No	No	Yes	Yes
12	M	61	NF-PitNET	No	2	81	Yes	No	Yes	Yes
13	M	51	ACTH secreting-PitNET	No	First: 1 Second: 2	28	Yes	No	Yes	Yes
14	M	31	NF-PitNET	No	2	41	Yes	No	No	No
15	F	66	NF-PitNET	No	2	140	Yes	No	No	Yes
16	M	48	NF-PitNET	Yes	2	82	Yes	No	Yes	No
17	M	63	NF-PitNET	Yes	2	40	No	No	Yes	No

Intraoperative CSF leaks occurred in 41 patients (43.6%). Sixteen patients (17%) experienced early postoperative complications (< 30 days), including CSF leak requiring reoperation (*n* = 7), meningitis (*n* = 3), brain hematoma (*n* = 2), postoperative hydrocephalus requiring ventriculoperitoneal shunting (*n* = 2), cerebral abscess (*n* = 1), and cavernous internal carotid artery injury (*n* = 1).

The patient with ICA injury underwent surgery for a recurrent clival chordoma with extensive involvement of the right clivus. The surgical field was severely distorted by scarring from prior surgery and radiation. During tumor removal, the right ICA was inadvertently lacerated.

Medpor was used only as a temporary compressive material (together with Spongostan, cotton wool, and sealant) to control bleeding. The patient subsequently underwent endovascular occlusion of the injured vessel.

### Re-operated patients (*n* = 25)

**Fig. 2:**
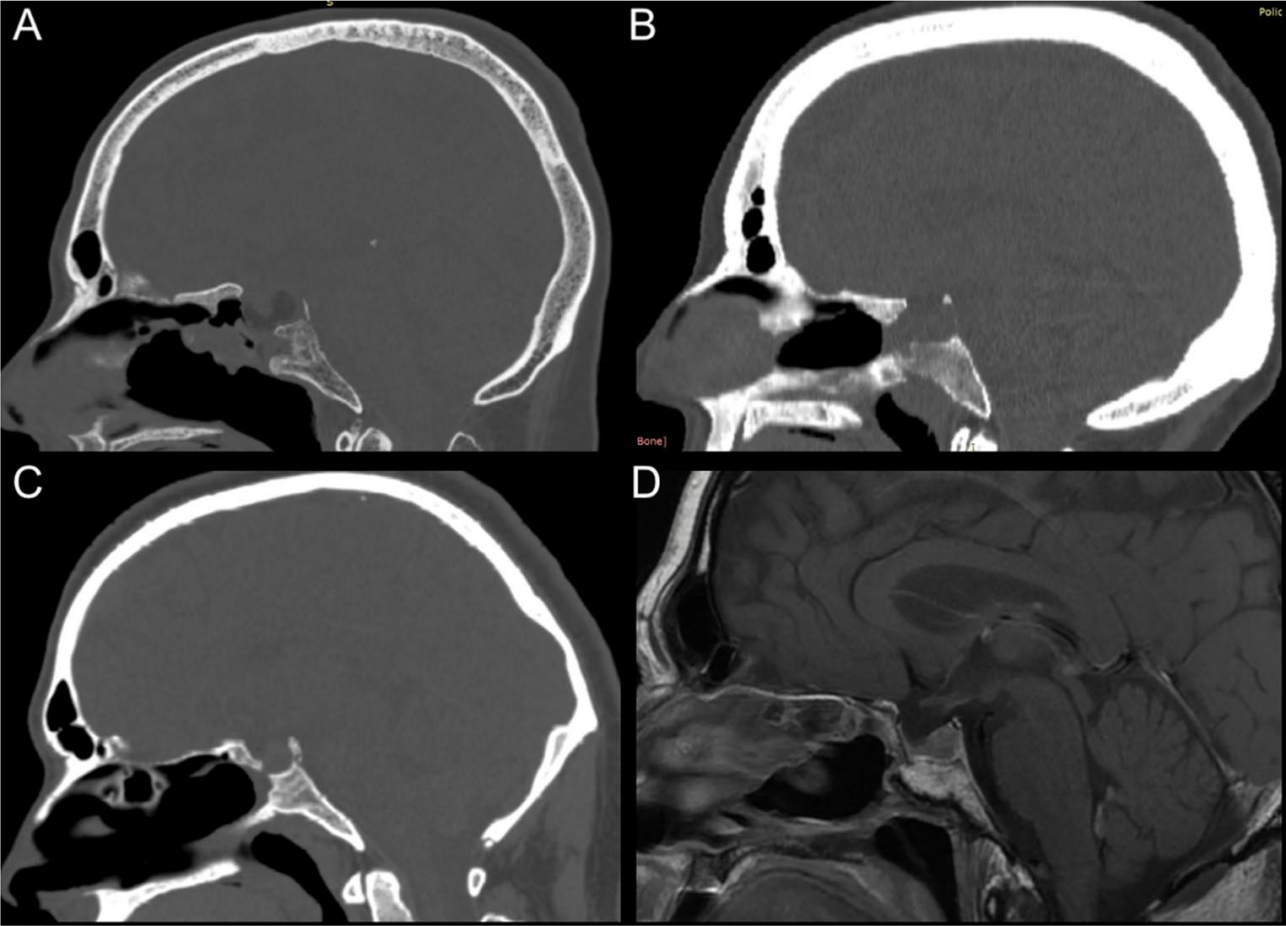
Radiological evaluation of osteointegration. Medpor is usually well recognized in MR imaging (D) as hypointense in T1- and T2 weighted images, while it is less evident in CT images, being hypo- or isodense. **A**, **B**: CT scans performed respectively 7 and 3 years after EEA with Medpor and nasoseptal: no osteointegration was identified. **C**; CT scan documenting partial osteointegration of Medpor 4 years after surgery. **D**: Brain MRI performed 7 years after endoscopic transnasal surgery with septal flap positioned: Medpor appears well osteointegrated, as confirmed intraoperatively

Twenty-five patients (26.6%) required reoperation for reasons including tumor recurrence (*n* = 16), CSF leaks (*n* = 5), postoperative hemorrhage (*n* = 2), and Medpor extrusion (*n* = 1). Seventeen of these reoperations occurred at least 6 months post-implantation (mean, 68 months; range, 24–144 months), allowing for evaluation of osteointegration. In these 17 patients, a nasoseptal flap was not used initially in 58.8% of cases; osteointegration was evident in only one patient, who had undergone the first surgery seven years prior with a nasoseptal flap (Fig. 2).

One patient complained of a chronic inflammatory/granulomatous sphenoidal reaction three years after a second surgery for a recurrent non-functioning PitNET. The patient complained of severe chronic nasal congestion and multiple episodes of epistaxis. An endoscopic nasal exploration revealed a septal perforation with an exuberant inflammatory scar tissue surrounding a partially extruded/non-osteointegrated Medpor implant, which was removed. No signs of infection, and no evidence of CSF leak were found. The subsequent stable resolution of symptoms was observed after 8 years of follow-up.

**Fig. 3:**
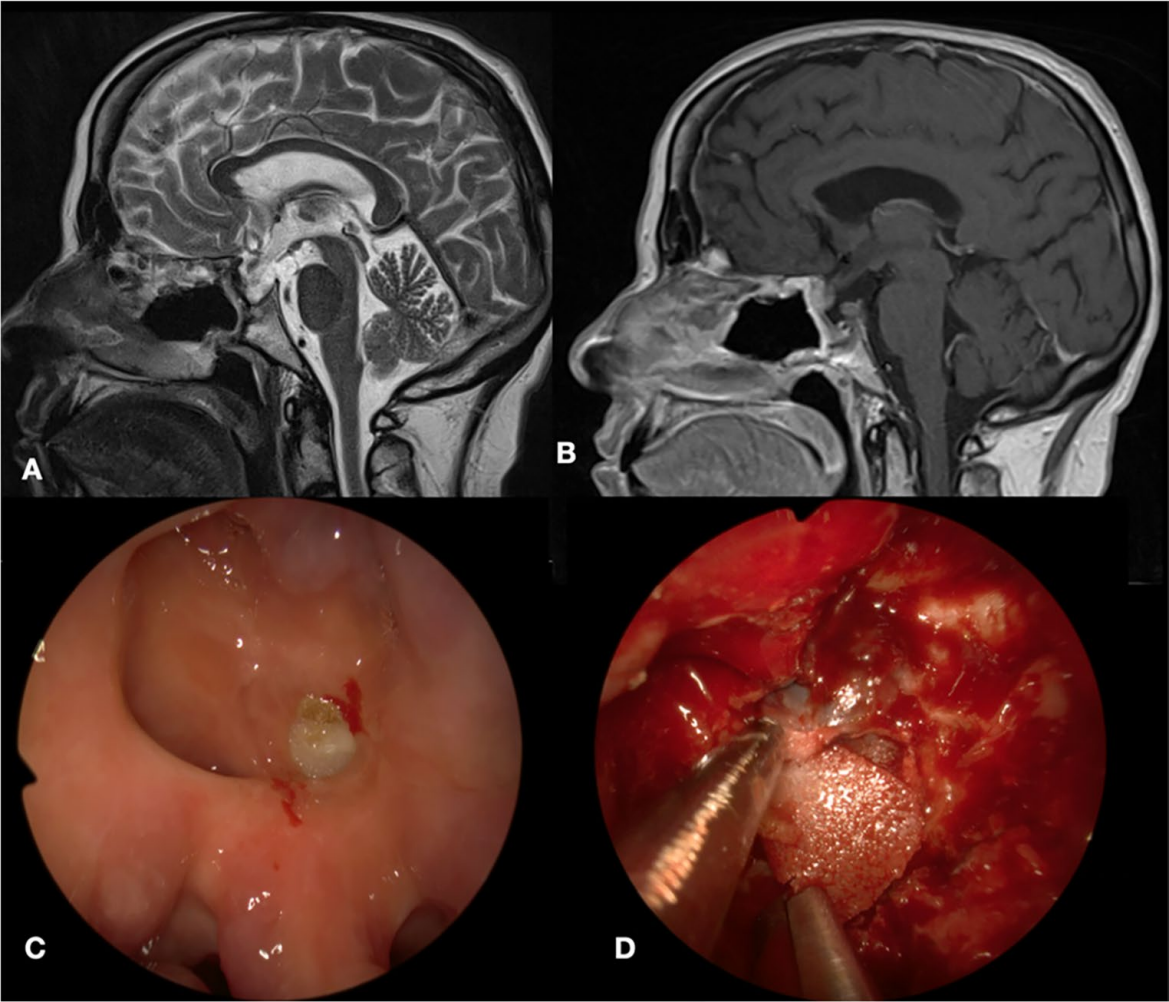
Intraoperative findings. **A**, **B**: Pre-operative T2 and T1 weighted brain MRI showing the ipointense Medpor. **C**: Intraoperative images with evidence of Medpor exposure through a mucosal defect. **D**: Intraoperative image of Medpor’s removal

Another case of macroscopically evident Medpor exposure found intraoperatively is described in Fig. 3.

The patient was a 66-year-old woman operated in 2012 for a NF-PitNET with left cavernous sinus invasion. Sellar floor reconstruction was performed using Medpor, dural sealant and nasoseptal flap.

Twelve years later a second EEA was performed for pathology recurrence and intraoperatively a septal perforation with an extruded Medpor implant were found.

### Non-reoperated patients (*n* = 69)

For the 69 patients who did not require reintervention, clinical follow-up (mean, 62.5 months; range, 1–144 months) and structured telephone interviews were conducted to assess outcomes. Follow-up beyond 24 months was achieved in 75% (52/69), with chronic nasal symptoms reported in 13.4% (7 of 52 with complete follow-up data); however, none required additional surgery or removal of Medpor. No implant-related severe complications or removals were reported among non-reoperated patients.

## Discussion

Effective reconstruction of skull base (SB) defects after endoscopic endonasal approaches (EEA) is crucial for minimizing postoperative complications, especially cerebrospinal fluid (CSF) leaks, which remain the most common problem in these surgeries [5, 9, 24]. An ideal reconstruction method should offer durable structural support, biocompatibility, low infection rates, and minimal inflammatory response while effectively sealing CSF pathways. Autologous grafts, vascularized flaps, and synthetic materials are used for this purpose, with porous high-density polyethylene (Medpor) increasingly adopted due to its structural stability and ease of handling during surgery to replace the bony layer [4, 8, 13, 19, 22–24, 27].

Medpor is frequently used in craniofacial and ENT reconstructive procedures, with a growing body of literature highlighting both its advantages and disadvantages [9, 10, 12, 13, 18, 22, 25, 28]. Despite its widespread application, the long-term behavior of Medpor implants in SB reconstruction, particularly regarding osteointegration within the dynamic sinonasal environment, remains insufficiently characterized. Our study, which integrates a systematic literature review with an institutional case series, provides updated insights into the long-term safety and osteointegration outcomes of Medpor implants in EEA skull base reconstructions.

Our institutional series showed a 94% rate of absent osteointegration in patients who required reoperation at least 6 months after implantation, as supported by preoperative CT scans and intraoperative observations. (Fig. 2)

Conversely, a systematic review of the literature [9–13, 16, 18, 22, 25, 28, 30] indicates that, out of 471 patients, poor osteointegration of the porous polyethylene implant in skull base reconstructions was documented in only 3 cases [12, 13]. One case was identified intraoperatively four years after the initial surgery, while the other two were documented on MRI after a median follow-up of 22 months. In their study, Park et al. [22] routinely performed postoperative MRI at 3 and 15 months post-surgery. Sellar reconstruction with Medpor was described as showing low-intensity signals on T1-weighted images, and no signs of Medpor dislocation or migration were observed during follow-up.

The discrepancy between the present case series and existing literature may stem from the fact that many included studies did not systematically use CT scans to confirm osteointegration or lacked intraoperative data on osteointegration during revision surgeries. In the present series, only one case demonstrated clear Medpor osteointegration on both preoperative imaging and intraoperative inspection. Notably, the second surgery occurred seven years after the first, and a nasoseptal flap had been used during the initial surgery, suggesting a potential role for vascularized nasoseptal flaps in Medpor osteointegration. However, even the use of the nasoseptal flap does not appear to have had a significant impact on osteointegration, considering that it was actually used in only 7 out of 17 cases, suggesting that other factors may have a more significant influence on the process.

Concerning post-operative complications, the systematic review reported that 5.7% (27/471) of patients experienced complications, mainly represented by CSF leaks (2.9%), followed by sinus infections, sinus inflammation, and meningitis. The institutional case series had a higher early complication rate (16 of 94 patients) experienced complications within 30 days of surgery, including six CSF leaks, three cases of meningitis, and a cerebral abscess.

This higher rate of early complications in the case series may reflect the use of Medpor in more complex cases where other reconstructive options were limited. In routine practice, cartilage or bone from the nasal septum was typically used to reconstruct the bony layer of the skull base reconstruction. When these options are not available, Medpor is an excellent solution, as it is easily positioned due to its limited thickness and the central extrusion that acts as a handle.

Regarding chronic sinusitis, Medpor-related with a need for second surgery for implant removal, three out of 471 (0.6%) patients are reported in the literature, which is in line with our institutional experience (1/95, 1%).

Ultimately, osteointegration is a multifactorial process affected by age, medical comorbidities, tobacco use, and lifestyle. Chronic sinusitis, mucosal inflammation, and mechanical stress—especially during postoperative recovery—can also affect osteointegration and increase the likelihood of extrusion [13]. The use of other synthetic materials, such as sealants, may also impact osteointegration and increase the rate of postoperative CSF leaks by impairing healing between the implant and surrounding tissues [6, 20, 29]. Further studies are needed to determine whether Medpor consistently leads to low long-term osteointegration rates (< 8%) in skull base reconstructions.

### Study limitations

This study has limitations, including its single-center retrospective design, potential underreporting of minor complications, and lack of standardized quantitative assessment of osteointegration across the included studies. The relatively small sample size of reoperated patients limits the generalizability of the osteointegration findings. Additionally, factors such as smoking, diabetes, and local sinonasal conditions, which may affect integration and complication rates, were not systematically analyzed.

The systematic literature review is limited by the inclusion of case reports and studies with short or incomplete follow-up. Additionally, osteointegration was not consistently assessed—either through CT imaging or surgical exploration—across the reviewed studies. This inconsistency may have led to an overestimation of osteointegration rates and complicates direct comparison with the present case series, which reflects a single-center experience with a standardized reconstruction technique and a limited number of cases.

## Conclusions

Medpor implants are safe and effective for skull base reconstruction following EEA, with low rates of implant-related complications and acceptable clinical tolerance during long-term follow-up. However, their osteointegration appears rare and limited on long-term follow-up. Therefore the implant functions primarily as an inert structural support rather than an integrating graft within the skull base.

We recommend that reconstruction of skull-base defects should prioritize modern vascularized flaps and autologous graft. These materials offer enhanced biological integration and have contributed to the significantly improved postoperative outcomes seen in contemporary endoscopic skull-base surgery.

Porous high-density polyethylene represents a valid option as a grafting material for skull base reconstruction, especially when autologous reconstruction is not feasible. Careful patient selection and tailored reconstructive planning are crucial for optimizing outcomes in endoscopic skull base surgery.

## Data Availability

No datasets were generated or analysed during the current study.

## Abbreviations

AC: Arachnoid Cyst
Ch. Hern: Chiasmal Herniation
Chord.: Chordoma
Chondros.: Chondrosarcoma
CPh.: Craniopharyngiomas
CS: Cavernous Sinus
CSF: Cerebrospinal Fluid
EEA: Endoscopic Endonasal Approach
EEEA: Extended Endoscopic Endonasal Approach
FESS: Functional Endoscopic Sinus Surgery
Germ.: Germinoma
Hyp.: Hyperplasia
NS: Nasoseptal
PitNET: Pituitary Neuroendocrine Tumor
Pneumoc.: Pneumocephalus
RCC: Rathke cleft cysts
SB: Skull Base
SCO: Spindle Cell Oncocytoma
SLA: Sublabial Approach
TSA: Transnasal Transsphenoidal Surgery
XGA: Sellar xanhogranulomas
